# A super resolution generative adversarial networks and partition-based adaptive filtering technique for detect and remove flickers in digital color images

**DOI:** 10.1371/journal.pone.0317758

**Published:** 2025-05-12

**Authors:** Thangavel Shanmugaraja, Natesapillai Karthikeyan, Subburathinam Karthik, Balamurugan Bharathi

**Affiliations:** 1 Department of Electronics and Communication Engineering, KPR Institute of Engineering and Technology, Coimbatore, Tamil Nadu, India; 2 Department of Computer Science and Engineering, SNS College of Technology, Coimbatore, Tamil Nadu, India; 3 Department of Computer Science and Engineering, Sri Ranganathar Institute of Engineering and Technology, Coimbatore, Tamil Nadu, India; Shanghai Jiao Tong University, China

## Abstract

Eliminating flickering from digital images captured by cameras equipped with a rolling shutter is of paramount importance in computer vision applications. The ripple effect observed in an individual image is a consequence of the non-synchronized exposure of rolling shutters utilized in CMOS sensor-based cameras. To date, there have been only a limited number of studies focusing on the mitigation of flickering in single images. Furthermore, it is more feasible to eliminate these flickers with prior knowledge, such as camera specifications or matching images. To solve these problems, we present an unsupervised framework Super-Resolution Generative Adversarial Networks and Partition-Based Adaptive Filtering Technique (SRGAN-PBAFT) trained on unpaired images from end to end Deflickering of a single image. Flicker artifacts, which are commonly caused by dynamic lighting circumstances and sensor noise, can severely reduce an image’s visual quality and authenticity. To enhance image resolution SRGAN is used, while Partition based Adaptive Filtering technique detects and mitigates flicker distortions successfully. Combining the strengths of deep learning and adaptive filtering results in a potent approach for restoring image integrity. Experimental results shows that the Proposed SRGAN-PBAFT method is effective, with major improvements in visual quality and flicker aberration reduction compared to existing methods.

## 1. Introduction

Applications like object recognition and medical image analysis can be improved by using the abundance of detailed information in high-resolution images [1]. The ability of neural networks to comprehend the mapping relationships between high-resolution and low-resolution images is essential in the super-resolution reconstruction of images. As deep learning has progressed, model parameters have gradually risen, and the network topology has grown increasingly complicated [2]. The power grid quasi-periodically alters AC-powered light sources’ luminance, resulting in rolling shutter camera images under artificial lighting frequently containing flickers [3]. More specifically, during exposure, varying intensities of quasi-periodic sinusoidal signals disturb the pixels captured in the picture. Worse yet, it might interfere with more advanced tasks that come next, such as position estimation, object detection, and interior scene recognition.

To tackle this problem, the majority of current approaches utilize historical data from lighting systems [4] or depend on imaging device settings, such as the inter-row delay of the rolling shutter [5], for the creation of digital filters aimed at mitigating flickering. However, because this background data is unavailable, they can’t be used in the real world. Another field of study looks at the analysis of image collections, namely the blank spaces within them. For example, combining images taken at different exposure times is one approach to developing a flicker suppression algorithm for images with a wide dynamic range [6]. This technique works particularly well for mitigating flicker in short-exposure images. It’s crucial to remember that this method cannot eliminate flicker from a single image because it requires numerous images of the same scene. However, images with complex backgrounds significantly degrade their performance, limiting their utility.

Data-driven methods have recently demonstrated impressive results in various image enhancement tasks. Supervised learning-based approaches for removing flicker become unfeasible due to challenges in obtaining flicker-free image pairs in real-world scenarios. Using unsupervised techniques provides an alternative solution. Since the generation and removal of flicker in images involve cyclic mapping complexities, we leverage the SRGAN’s ability to facilitate information transfer between source and destination domains, enabling the development of a single-image DEFlickering technique and vice versa. However, the SRGAN generator introduces distortions in color and structure that must be addressed, as it cannot eliminate flickers without considering their patterns. We have meticulously tailored the network topology to align with specific objectives to tackle these issues and have introduced new constraint function designs to regularize network training. Additionally, we employ highly trained discriminators in a unified system to detect flicker. According to the results of our experiments, our Deflickering algorithm outperforms common non-learning approaches and the older SRGAN when learning from synthetic and real images [7].

The algorithmic structure and design reasoning of PBAFT’s is explained by its theoretical basis. We analysis this method with multiple dataset and compare it to flicker reduction approach. PBAFT is required for satellite photography, medical imaging and cinematography in order to clarify its applications and restore image quality. This article helps to understand the mechanism of PBAFT and incorporate it into image processing pipelines for the compelling and authentic visual content creation.

- To enhance image resolution SRGAN is used and the flicker distortions were deducted and reduced by the PBAFT filtering technique.
- By combining these two method the strength of deep learning and adaptive filtering technique leverages to offer a powerful result for restoring image integrity.
- The results from the experimental validation reveals the proposed SRGAN-PBAFT method is effective and shows great improvements in visual quality and reduction in flicker abnormalities compared to the other existing methods.
- To upgrade multimedia content by simultaneously tackling resolution enhancement and flicker artifact reduction.

Following this structure, the rest of the paper will discuss: In Section 2, we present a synopsis of the relevant literature. The suggested system is described in Section 3, the experimental results are presented and discussed in Section 4, and the conclusion and future directions are discussed in Section 5.

## 2. Literature survey

The Section 2 discusses the literature survey of existing system, Nowisz et al. [8] offer a method for removing flicker from frame streams that exceed 200 frames per second and work as an online filter. The bulk of solutions in the literature emphasize efficacy and accuracy over speed of operation. Conversely, our initial method is optimized for speed while maintaining enough accuracy to be used before computing differential frames to identify motion in streams. Our method is flexible and performs well with various flickering light sources and when lighting circumstances change. The trial’s results demonstrate how well the CPU and GPU technology work to monitor items of interest in early applications of a fast-approaching badminton system available for acquisition.

Lin et al. [9] have described the DEFlicker Cycle GAN, an unsupervised architecture trained on unpaired images for end-to-end single-image DEFlickering. Flicker and gradient loss are two new capabilities that we carefully designed to decrease the possibility of color distortion and edge blurring. We also include a cycle-consistency loss to ensure that image content similarity is retained. Furthermore, we present a novel technique that employs the knowledge of two pre-trained Markovian discriminators in an ensemble approach to detect flicker artifacts in images. Extensive trials on synthetic and real datasets demonstrate that our DEFlicker Cycle GAN exhibits strong generalization capabilities and achieves high accuracy in flicker detection, outperforming a well-trained ResNet50 classifier. It is also very good at reducing flicker within a single image.

Gao et al. [10] developed a groundbreaking two-stage detection technique that greatly enhanced detection speed for addressing adaptable targets. However, the robustness of hypothesis testing using the K distribution is compromised by time-varying clutter. Additionally, the dynamic programming-based Track before Detect method encounters challenges in multi-target detection due to the absence of prior knowledge, necessitating higher processing overhead. Moreover, target state disruptions occur due to the target’s flickering and blanking conditions. The cumulative integration quantity of the previous technique must be increased, resulting in reduced performance in flickering multi-target detection. We introduce a novel method for low Signal-to-Clutter Ratio (SCR) flickering multi-target detection, which can be attributed to its straightforward architecture and minimal data requirements.

Zhang et al. [11]’s innovative multi-frame joint detection method is the Biological Memory Model-based Multi-Target Joint Detection Technique. This approach locates elusive, intermittent items on rivers and seas. They solved multi-target identification problems in unexpected target states with a pre-filtering operator. BMM-DP-MJD’s memory weight-based integration ensures accurate identification of flickering dynamic targets. When the Signal-to-Clutter Ratio (SCR) was low, 3 to 8 dB, flashing targets improved detection accuracy in simulations. Experimental results show that this approach accurately locates ships and micro buoys in river environments, enhancing marine navigation radar target detection.

Chudasama et al. [12] presented two unique and effective computational algorithms for single-image super-resolution that reduce the component count to improve performance. While some hyperparameters were necessary, decreasing the model’s parameters might shorten the time needed to tune them. To reduce the time spent making changes, the technique took a different tack, employing optimization algorithms to find a variety of model hyperparameter combinations on their own. There are a few general model hyperparameter search approaches provided.

Kopania et al. [13] framework the system’s architectural progress. The shuttlecock was initially tracked in three dimensions by cameras above and around the lines. An enhanced version used cameras throughout the court instead of three-dimensional reconstruction. This study compares our system’s competition results to linesmen’s decisions. In badminton events, our technology matches the world’s largest commercial goods in accuracy and processing speed. Our design and algorithms make installation faster and easier, making our system more pervasive, robust, adaptive, and adjustable to sporting facility needs.

Shekhar et al. [14] demonstrate that per-frame stylised videos can be temporally coherent regardless of frame stylization. Reduce the gradient domain difference to retain per-frame processed output similarity. Interactive consistency management provides faster and more accurate optical flow computation on the incoming video stream for stabilization than previous methods. For fast optical-flow inference, we use PWC-Net-based lightweight flow networks. Real-time HD frame rates are achieved using GPU-based optimization. User study shows that our temporally consistent output beats alternative methods.

Si et al. [15] introduced the IA multi-target filter, which simultaneously approximations the multi-target state and the clutter distribution, while characterizing unidentified clutter as a gamma distribution. However, it continues to work on the basis of a known cluster distribution, a limitation that may be more successful in practice. The SRBE-PF-TBD technique is notable for its versatility and durability, since it works well even without prior clutter information and consistently yields great detection performance in a variety of target identification conditions. Unfortunately, due to its high processing cost and theoretical base in particle filtering, this approach is inappropriate for real-world deployments.

Raihan et al. [16] compared underwater image restoration methods. Earlier methods used hardware like polarizers, sensors, lasers, etc. To capture images of the same topic and run them through an algorithm to sharpen them. That hardware approaches involve complex hardware setups and are more expensive. For underwater images improvement with low visibility, computer vision algorithms and optical models have been created. DCP, histogram equalization, and color correction are popular. The literature also shows that optical images restoration works. Wavelength correction and artificial light detection and exclusion work well together.

### 2.1. Limitations for existing system

- A potentially leading method need to be developed to treat specific types of flickers which leads to insufficient detection or eradication.
- Detecting and removing flickers in complex scenarios, where different portions of the image exhibit varying flicker characteristics, can be challenging.
- Digital images are often susceptible to noise, which might be misinterpreted as flickers, resulting in false positive detection.
- Flickers can occur at varied rates and phases across frames due to temporal synchronization.
- Due to dynamic lighting conditions, camera movements and object interactions flicker characteristics can change over time.

## 3. Proposed system

**Fig 1:**
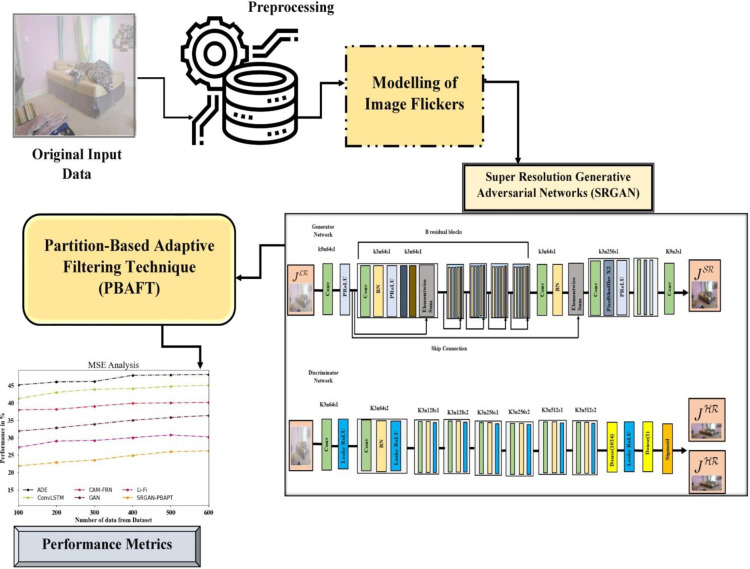
Proposed method of SRGAN-PBFPT.

The Section 3 discusses the proposed method about an unsupervised system SRGAN-PBAFT which trained on a unpaired image to perform deflickering from start to finish in a single image. There are several factors which cause Flicker artifacts including dynamic illumination condition and sensor noise can minimize an image visual quality and trustfulness. This proposed work introduced to enhance image resolution and mitigates flicker distortions effectively using SRGAN-PBAFT. To produce a powerful solution to restore image integrity we combine these two algorithms. The block diagram illustrating the SRGAN-PBAFT method is displayed in Fig 1.

### 3.1. Datasets

**Fig 2:**
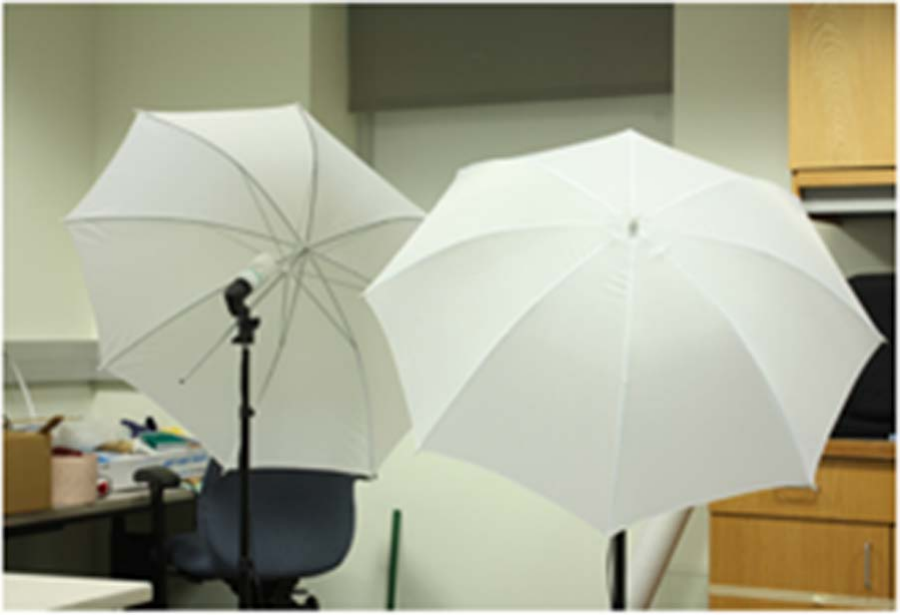
Sample images NYU2 Depth dataset.

**Fig 3:**
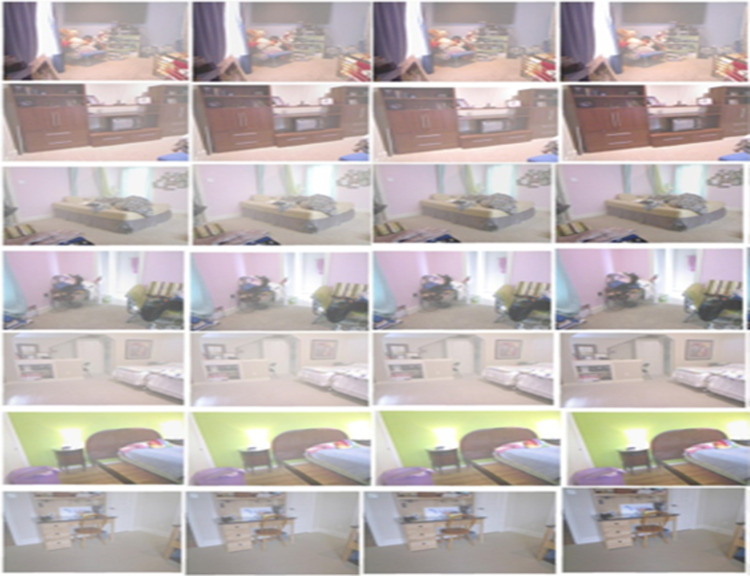
Sample images Middlebury stereo dataset.

**Fig 4:**
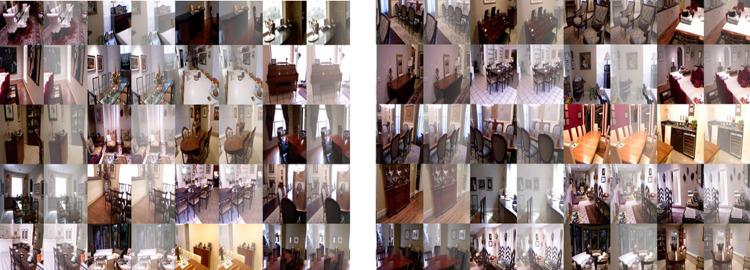
Sample images SOTS dataset.

Utilizing a haze-free image and the corresponding depth map downloaded from the NYU2 Depth dataset, create synthetically hazy images as explained in [17]. In this procedure, compute the transmission map for each image, represented as t(x, y), by utilizing Equation (2), which employs the scattering coefficient (β) and the depth information (d(x, y)). Then, to combine the light from different sources, we use a model of air scattering. (A), the transmission map (t(x, y)), and the haze-free image in order to generate the hazy image. This paper makes the assumption of globally uniform ambient light (A) in our method. We select β, the scattering coefficient, from the set {0.4, 0.6, 0.8, 1.0, 1.2, 1.4, 1.6}, and set [a, a, a] as the ambient light A, with ‘a’ ranging between [0.7, 1.0]. For assistance with this, we select 1,000 images from the NYU2 Depth dataset that are clear of fog at random. Fig 2 shows some examples of the photos found in the NYU2 Depth dataset. Generate hazy images by using atmospheric light A and randomly sampled scattering coefficient β, resulting in ten training images for each haze-free image. In total, 10,000 training images are available. This data create a 300-image indoor test synthetic dataset using the imagery and depth maps from the Middlebury stereo dataset, as shown in Fig 3 [18]. Additionally, as an external synthetic test dataset, Fig 4 utilizes 500 outdoor synthetic hazy images taken from the SOTS [19] dataset. It’s important to note that none of these test images are used during the training phase. This research ensures ethical AI development by using responsibly sourced data, minimizing biases, and preventing misuse in deceptive image manipulation. This research prioritize transparency, privacy protection, and energy-efficient model training to promote fairness and sustainability in digital image processing.

### 3.2. Pre-processing

Several essential techniques can be employed to preprocess digital color images to detect and remove flickers. Image scaling and color space conversion may first be used to ensure uniformity and convenience of processing. Histogram equalization can enhance image contrast, aiding in the identification of flickers. Noise reduction techniques, such as Gaussian or median filtering, can help mitigate minor fluctuations. Additionally, frame differencing and motion detection algorithms can be implemented to identify flickering regions within the image. These preprocessing steps lay the foundation for accurate flicker detection and removal, improving the overall quality and stability of digital color images.

### 3.3. Modelling of image flickers

Images with sinusoidal impulses but lack flicker can be called flickering images [20]. Flicker can be represented as follows because of its sinusoidal-like pattern:


$$X^{c}(a,b)=Y^{c}(a,b)+A^{c}\cos(4\pi \frac{f_{ENF}}{f_{row}}a+\varphi ),c\in \{R,G,B\}$$ (1)

**Fig 5:**
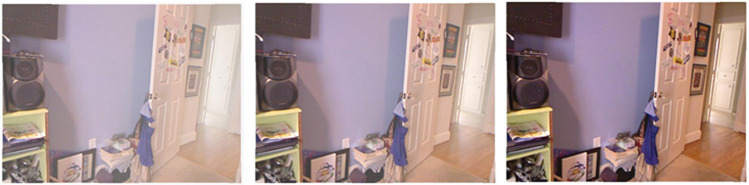
Flicker signals images.

The color channel is indicated by ‘c,’ and the image’s pixel coordinates are signified by ‘(a, b).’ ‘X’ signifies a flickering image, while ‘Y’ defines the image without flicker. The flicker in channel ‘c’ has an intensity of ‘Ac.’ Fig 5 shows that flicker signals can have varying intensities in each RGB channel because the light source’s spectrum affects each channel’s flicker intensity. In a rolling shutter camera, the electric network frequency (ENF), usually 50 Hz or 60 Hz, is represented as $f_{ENF}$, while the row sampling frequency is indicated as $f_{row}$. Notably, the flicker signal frequency is double that of the typical ENF due to the power consumption of lighting fixtures. The first row of image X is captured simultaneously as the power grid’s first phase begins. The goal of image deflickering is to replace a flickering image (X) with a steady image (Y).

### 3.4. Super resolution generative adversarial networks

From a low-resolution input image $J^{LR}$, super-resolved image I SR was obtained. The low-resolution equal of $J^{HR}$ high-resolution counterpart is represented here as $J^{LR}$.

The high-resolution images can be accessed exclusively during the training phase. These $J^{LR}$ images are generated for training purposes through the application of a Gaussian filter $J^{HR}$ and a downsampling process with a downsampling reason denoted as ‘r.’ When dealing with an image possessing C color channels, we employ the notation ‘rW×rH×C’ to represent it and utilize a real-valued tensor with ‘W×H×C’ and ‘$J^{HR}$’ dimensions.

This paper objective is to train a generating function G that computes the appropriate HR image for a given LR input image. Then, train a generator network as a feed-forward CNN parameterized by $\theta_{G}$ in order to accomplish this. Here, $G_{\theta_{G}}$ which is achieved by $L^{SR}$ optimization, a loss function unusual to SR. We solve the following for training images $J_{n}^{HR},n=1,....,N$ with appropriate $J_{n}^{LR},n=1,....,N$:


$$\hat{\theta_{G}}=\arg\underset{\theta_{G}}{\min}\frac{1}{N}\sum_{n=1}^{N}L^{SR}(G_{\theta_{G}}(J_{n}^{LR}),J_{n}^{HR})$$ (2)

Specifically, a weighted sum of many loss components corresponding to different needed elements of the recovered SR image will be used to compute a perceptual loss $L^{SR}$ in this study.

### 3.5. Adversarial network architecture

To tackle the difficulty of addressing the adversarial min-max problem, this study presents a discriminator network called $D_{\theta_{D}}$, and uses $G_{\theta_{G}}$ to optimize it alternately:


$$\begin{array}{l} \underset{\theta_{G}}{\min}\underset{\theta_{D}}{\max}E_{J^{HR∼P_{train}}(J^{HR})}[\log D_{\theta_{D}}(J^{HR})]+\\ E_{J^{LR∼PG}(J^{LR})}[\log(1-D_{\theta_{D}}(G_{\theta_{G}}(J^{HR}))] \end{array}$$ (3)

**Fig 6:**
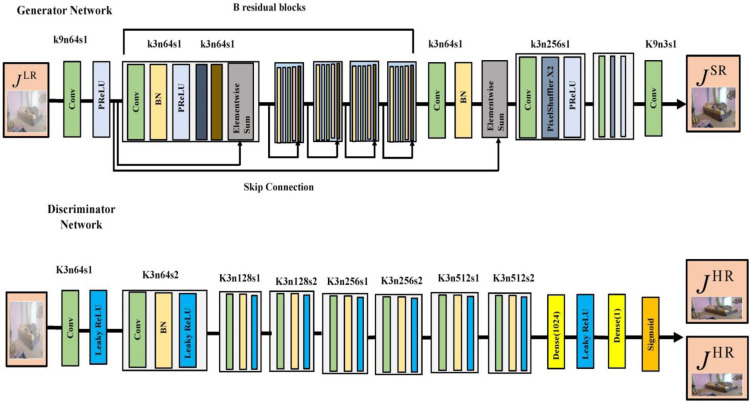
Architecture of generator and discriminator networks.

A differentiable discriminator (D) can be instructed to distinguish between real and super-resolved images, but a generative model (G) can be built to outperform D. Using this method, our generator is able to produce outputs that are highly realistic, making them difficult for D to classify. This, in turn, confirms that solutions are located in the subspace or manifold of natural images and have better perceptual properties. The mean square error (MSE) and other pixel-wise error metrics yield less accurate solutions. Fig 6 show that our generator network G, intended for deep water, comprises B identical residual blocks. Train a discriminator network to differentiate between generated SR data and actual HR images. In accordance with the design objectives, we employ the LeakyReLU activation function (with α set to 0.2) to prevent max-pooling throughout the network. The maximization problem described in Equation (3) is integrated into the discriminator network. Following this, two dense layers are added, and a final sigmoid activation function is applied after the 512 feature maps to yield a probability for straightforward categorization.

### 3.6. Perceptual loss function

The performance of the generator network is impacted by the definition of our perceptual loss function. Although Mean Squared Error is widely used to approximate low-resolution (LR) images, we improve on previous research [21–22] by developing a loss function that assesses solutions according to perceptually meaningful features. The adversarial loss component plus the content loss $L_{X}^{SR}$ are the weighted sums that make up the perceptual loss.


$$L^{SR}=\underset{Perceptual\;loss(for\;PBAPT\;content\;losses)}{\underset{︸}{\underset{content\;loss}{\underset{︸}{L_{X}^{SR}}}+\underset{adversarial\;loss}{\underset{︸}{10^{-3}L_{Gen}^{SR}}}}}$$ (4)

The possibilities for the adversarial loss $L_{X}^{SR}$ Gen and the tent loss $L^{SR}$ are described below.

#### 3.6.1. Content loss.

The following formula is used to determine the Mean Squared Error (MSE) loss per pixel:


$$L_{MSE}^{SR}=\frac{1}{r^{2}WH}\sum_{x=1}^{rW}\sum_{y=1}^{rH}\left(J_{x,y}^{HR}-G_{\theta_{G}}{\left(J^{LR}\right)}_{x,y}\right)$$ (5)

This imaginary optimization aim is used the most, and it forms the basis for many inventive approaches. Solutions to MSE optimization difficulties often need more high-frequency content, even at very high PSNRs, leading to perceptually unsatisfactory solutions with overly smooth textures.

In this case, the PBAPT network’s $W_{i,j}$ and $H_{i,j}$ feature maps’ dimensions are described.

#### 3.6.2. Adversarial loss.

This paper supplements the perceptual loss with the generative constituent of our GAN in addition to the previously mentioned content losses. Using the discriminator $D_{\theta_{D}}$ ($G_{\theta_{G}}$ ($I^{LR}$)) probabilities across all training samples, the generative loss l SR Gen is defined as


$$G_{en}^{SR}=\sum_{n=1}^{N}-\log D_{\theta_{D}}(G_{\theta_{G}}(I^{LR}))$$ (6)

The likelihood that the reconstructed image $G_{\theta_{G}}(I^{LR})$ is a genuine HR image is represented here by $D_{\theta_{D}}(G_{\theta_{G}}(I^{LR}))$. Rather of minimizing $-\log D_{\theta_{D}}(G_{\theta_{G}}(I^{LR}))$, we minimize $\log[1-D_{\theta_{D}}(G_{\theta_{G}}(I^{LR}))]$ for improved gradient behaviour.

### 3.7. Partition-based adaptive filtering technique (PBAFT)

In various contexts, from entertainment to medical imaging, digital color images are essential. However, they frequently experience flickers, which can be caused by several factors such as sensor noise, compression errors, or abrupt changes in lighting. Flickers are abrupt, erratic changes in pixel values that give the appearance that an image is unstable and inconsistent. With its effective approach to flicker detection and eradication, the Partition-Based Adaptive Filtering Technique represents a significant advancement in image processing [23]. This approach effectively locates and minimizes flicker-induced disruptions by applying adaptive filtering inside different image segments.

Consider an unknown linear system.


$$k\left(m\right)=r^{T}\left(m\right)w^{o}+s\left(m\right)$$ (7)

$r\left(m\right)=\left[r\left(m\right),...,r{\left(m-L+1\right)}^{T}\right]$ where $w^{o}\in {\mathbb{R}}^{L\times 1}$ signifies the unknown system vector. For the input vector; $r\left(m\right)$ stands for the input signal at instant m; $k\left(m\right)$ stands for the corresponding desired output signal; and $s\left(m\right)$ stands for observation noise. Next, the error signal $e\left(m\right)$ is explained as


$$e\left(m\right)=k\left(m\right)-r^{T}\left(m\right)w\left(m\right)$$ (8)

The estimate of two at instant n is given by w(n). We may change the PBAPT algorithm’s updating formula.


$$w\left(m+1\right)=w\left(m\right)+\sigma e\left(m\right){\left[\left|r\left(m\right)\right|\right]}^{\beta }o\;sign\left(r\left(m\right)\right)$$ (9)

Where *σ* is the step-size parameter and 0<β≤1 is satisfied by the fractional-order α. The PBAFT will degenerate to the conventional LMS algorithm upon β=1.

We examine the PBAFT algorithm’s transient and steady-state performance in terms of mean-square error. We define a diagonal matrix below to make the following mathematical formula simpler:


$$K\left(m\right)△\;diag\left\{\left|r\left(m\right)\right|,\left|r\left(m-1\right)\right|,.....,\left|r\left(m-L+1\right)\right|\right\}$$ (10)

As a result, the updating formula in (9) can be reformulated as


$$\begin{array}{l} w\left(m+1\right)=w\left(m\right)+\sigma e\left(m\right)K^{\beta }\left(m\right)sign\left(r\left(m\right)\right)\\ \;=w\left(m\right)+\sigma e\left(m\right)K^{\beta -1}\left(m\right)\left(r\left(m\right)\right) \end{array}$$ (11)

After that, the weight-related error vector is defined as follows:


$$\tilde{w}\left(m\right)△\;w^{o}-w\left(m\right)$$ (12)

The formula for updating the weight error vector could be obtained by substituting equation (12) for equation (11).


$$\tilde{w}\left(m+1\right)=\tilde{w}\left(m\right)-\sigma e\left(m\right)K^{\beta -1}\left(m\right)r\left(m\right)$$ (13)

The error in (8) can be reconsidered as (12).


$$e\left(m\right)=r^{T}\left(m\right)\tilde{w}\left(m\right)+s\left(m\right)=e_{b}\left(m\right)+s\left(m\right)$$ (14)

Where $e_{b}\left(m\right)=r^{T}\left(m\right)$ stands for an excess error that is noise-free. When we add (14) to (13), we get


$$\tilde{w}\left(m+1\right)=B\left(m\right)\tilde{w}\left(m\right)-\sigma s\left(m\right)K^{\beta -1}\left(m\right)r\left(m\right)$$ (15)

Where


$$B\left(m\right)=I_{L}-\sigma K^{\beta -1}\left(m\right)R\left(m\right)$$ (16)

Same, $R\left(m\right)=r\left(m\right)r^{T}\left(m\right)$. We use the subsequent two well-known hypotheses to help with analysis.

Assumption 1: The autocorrelation matrix

$\left\{EN\left[r^{2}\left(m\right)\right],EN\left[r^{2}\left(m-1\right)\right],....,EN\left[r^{2}\left(m-L+1\right)\right]\right\}$ is utilized to ascertain the autocorrelation matrix for the temporally independent random process that is the input signal $r\left(m\right)$.

Assumption 2: The noise, referred to as $s\left(m\right)$, is independent of $r\left(m\right)$ and is made up of zero-mean i.i.d random procedures with variance equal to $\sigma_{s}^{2}$.

#### 3.7.1. Mean square transient behavior.

By applying Assumptions 1 and 2 along with the weighted Euclidean norm expectations of (15), we may obtain


$$\begin{array}{l} EN\left[{\left\|\tilde{w}\left(m+1\right)\right\|}_{\Sigma }^{2}\right]=EN\left[{\left\|\tilde{w}\left(m\right)\right\|}_{T}^{2}\right]\\ \;+\sigma^{2}E\left[s^{2}\left(m\right)\right]EN\left[{\left\|K^{\beta -1}\left(m\right)r\left(m\right)\right\|}_{\Sigma }^{2}\right]\; \end{array}$$ (17)

Where $T=EN\left[B^{T}\left(m\right)\Sigma B\left(m\right)\right]$ denotes the weighted matrix and *Σ* denotes any symmetric semi-definite matrix. T can be vectorized using the Kronecker product property.


$$vec\left(T\right)=EN\left[B^{T}\left(m\right)⊗B^{T}\left(m\right)\right]vec\left(\Sigma \right)=P\theta$$ (18)

Where *θ* = $vec\left(\Sigma \right)$ and


$$\begin{array}{l} P=EN\left[B^{T}\left(m\right)⊗B^{T}\left(m\right)\right]\\ \;=I_{L^{2}}-I_{L}⊗\sigma EN\left[K^{\beta -1}\left(m\right)R\left(m\right)\right]\\ \;-\sigma EN\left[K^{\beta -1}\left(m\right)R\left(m\right)⊗I_{L}\right]\\ \;+\sigma^{2}EN\left[K^{\beta -1}\left(m\right)R\left(m\right)⊗K^{\beta -1}\left(m\right)R\left(m\right)\right]\\ \;\approx I_{L^{2}}-I_{L}⊗\sigma EN\left[K^{\beta +1}\left(m\right)\right]-\sigma EN\left[K^{\beta +1}\left(m\right)\right]⊗I_{L} \end{array}$$ (19)

For sufficiently small step-size *σ*, the approximation of (19) makes sense. The second term on (17)’s suitable half surface can therefore be further stated as


$$EN\left[s^{2}\left(m\right)\right]EN\left[{\left\|K^{\beta -1}\left(m\right)r\left(m\right)\right\|}_{\Sigma }^{2}\right]=\lambda_{s}^{2}{\left[vec\left(Q\right)\right]}^{T}\theta$$ (20)

Where


$$Q=EN\left[K^{\beta -1}\left(m\right)R\left(m\right)K^{\beta -1}\left(m\right)\right]=EN\left[K^{2\beta }\left(m\right)\right]$$ (21)

Thus, it is possible to rewrite (17) as


$$EN\left[{\left\|\tilde{w}\left(m+1\right)\right\|}_{\theta }^{2}\right]=EN\left[{\left\|\tilde{w}\left(m\right)\right\|}_{P\theta }^{2}\right]+\sigma^{2}\lambda_{s}^{2}{\left[vec\left(Q\right)\right]}^{T}\theta$$ (22)

Using (22) as an iteration starting with m =  0, we get


$$EN\left[{\left\|\tilde{w}\left(m+1\right)\right\|}_{\theta }^{2}\right]=EN\left[{\left\|\tilde{w}\left(0\right)\right\|}_{P^{n+1}}^{2}\right]+\sigma^{2}\lambda_{s}^{2}{\left[vec\left(Q\right)\right]}^{T}\sum_{i=0}^{m}P^{i}\theta$$ (23)

$EMSE\left(m\right)△EN\left[e_{b}^{2}\left(m\right)\right]$ the popular performance metric is defined as the excess mean-square error (EMSE). The theoretical EMSE can be constructed by selecting an appropriate $\theta =vec\left(Z_{r}\right)$.


$$\begin{array}{l} EMSE\left(m+1\right)=EMSE\left(m\right)+\sigma^{2}\lambda_{s}^{2}{\left[vec\left(Q\right)\right]}^{T}P^{m}vec\left(Z_{r}\right)\\ \;+{\left[\tilde{w}\left(0\right)⊗\tilde{w}\left(0\right)\right]}^{T}\left(P-I_{L^{2}}\right)P^{m}vec\left(Z_{r}\right) \end{array}$$ (24)

Likewise, by setting the parameter $\theta =vec\left(I_{L}\right)$, the mean square deviation learning curve may be calculated.

#### 3.7.2. Mean square steady-state behavior.

By using the value m→∞ from equation (22) and presumptuous that $EN\left[{\left\|\tilde{w}\left(m+1\right)\right\|}^{2}\right]=EN\left[{\left\|\tilde{w}\left(m\right)\right\|}^{2}\right]$ is in a steady-state condition, the limit may be computed.

Thus, the theoretic stable *θ* state EMSE can be achieved by inserting ${\left(I_{L^{2}}-P\right)}^{-1}vec\left(Z_{r}\right)$.

Also, we assume that the distribution of the input signal u(n) elements is the similar. As a result, we get $Q=EN\left[{\left|r\left(m\right)\right|}^{2\beta }\right]$, $P=I_{L^{2}}-2\sigma E{\left[{\left|r\left(m\right)\right|}^{\beta +1}\right]}_{I_{L^{2}}}$, where $\lambda_{r}^{2}$ denotes the $r\left(m\right)$ variance. As a result, the steady-state EMSE is different.


$$EMSE\left(\infty \right)=\frac{\sigma L}{2}\lambda_{s}^{2}\lambda_{r}^{2}{\left(EN\left[{\left|r\left(m\right)\right|}^{\beta +1}\right]\right)}^{-1}EN\left[{\left|r\left(m\right)\right|}^{2\beta }\right]$$ (25)

The two expectancies of (25) can be explicitly derived when the zero-mean Gaussian signal by using the formula


$$EN\left[{\left|r\left(m\right)\right|}^{\beta +1}\right]=\frac{{\left(\sqrt{2}\lambda_{r}\right)}^{\beta +1}}{\sqrt{\pi }}\Gamma \left(\frac{\beta +2}{2}\right)$$ (26)

$$EN\left[{\left|r\left(m\right)\right|}^{2\beta }\right]=\frac{{\left(\sqrt{2}\lambda_{r}\right)}^{2\beta }}{\sqrt{\pi }}\Gamma \left(\frac{2\beta +1}{2}\right)$$ (27)

Where $\Gamma \left(.\right)$ displays the Gamma function. Consequently, the steady-state EMSE of equation (21) can be expressed as


$$EMSE\left(\infty \right)=\frac{{\left(\sqrt{2}\right)}^{\beta -3}\Gamma \left(\frac{2\beta +1}{2}\right)}{\Gamma \left(\frac{\beta +2}{2}\right)}\sigma L\lambda_{s}^{2}\lambda_{r}^{\beta +1}$$ (28)

To further simplify the steady-state Mean Squared Error (EMSE) of equation (25), one can extract r(m) from a uniform distribution whose mean is zero.


$$EMSE\left(\infty \right)=\frac{\beta +2{\left(\sqrt{3}\right)}^{\beta -1}}{4\beta +2}\sigma L\lambda_{s}^{2}\lambda_{r}^{\beta +1}$$ (29)

Partition-based adaptive filtering technique algorithm:

Initialization:

Define the input signal r[m] and desired signal k[m].

Choose the filter length L and partition size P.

Set the w[k] filter coefficients to small random values or zeros.

Set the *σ* (learning rate) step size parameter

Partition the Data:

Divide the input signal x[m] and desired signal k[m] into non-overlapping partitions of size P.

For instance, if the input signal has M samples, you will have M/P partitions.

Adaptive Filtering Loop

For each partition (i = 1 to M/P):

Extract the current partition of the input signal x[i] and desired signal k[i].

Perform the following steps within the partition

Step 1: Filtering Operation

Apply the filter defined by coefficients w[k] to the current partition of the input signal to generate an estimate y[i].

Compute the error signal e[i] = k[i] - y[i].

Step 2: Update Filter Coefficients:

Update the filter coefficients w[k] using an adaptive algorithm such as the LMS (Least Mean Squares) algorithm or NLMS (Normalized LMS):

w[k] =  w[k] +  *σ* *  e[i] *  x[i-k], for k =  0, 1, 2,..., L-1.

Step 3: Repeat until Convergence or Fixed Number of Iterations:

Repeat the adaptive filtering loop for each partition for a predetermined number of iterations or until the convergence requirements are satisfied (such as when the error becomes sufficiently reduced).

Output

The modified filter that minimizes the error across all partitions is represented by the final filter coefficients, w[k].

## 4. Result and discussion

**Fig 7:**
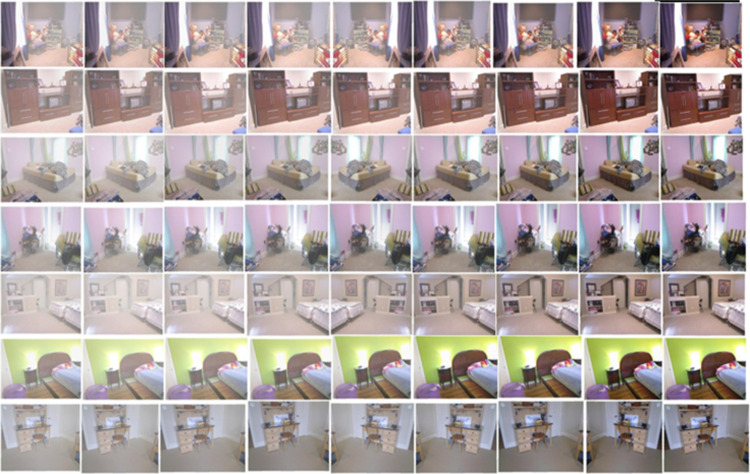
Images after flicker removing in NYU2 depth dataset.

**Fig 8:**
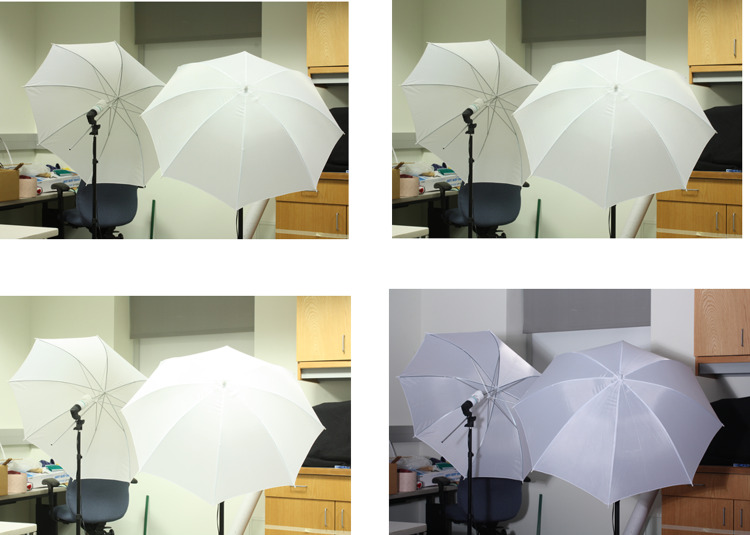
Images after flicker removing in Middlebury stereo dataset.

The Section 4 discuss the result of the experiment settings and performance evaluation, and compares the proposed system with existing systems like GAN (Generative Adversarial Networks) [24], convLSTM (Convolutional Long Short Term Memory) [25], CAM-FAR (class attention map-based flare removal network) [26], ADE (Adaptive Differential Equalization) [27], and Li-Fi (light-fidelity) [28]. Fig 7 shows the images after flicker removal in the NYU2 depth dataset, while Fig 8 shows the images after indication removal in the Middlebury stereo dataset.

### 4.1. Experiment settings

“We use an NVIDIA TITAN X GPU to train the networks. The MatConvNet toolkit is used to implement the suggested approach [29]. Every training image has been scaled to 320 x 240 pixels. Batch size, weight decay, and momentum have parameters of 10, 0.001, and 0.9, respectively. The ambient light module and the transmission map module have starting learning rates of 10−3 and 10−6, respectively. Moreover, after 20 epochs, both modules’ learning rates drop by a factor of 10. After 80 epochs, the training period comes to an end. The parameters are initialized with λt = 1, λA = 102, and λP = 5 × 10−4. The convolutional layer’s kernel size (h × w) in the atmospheric light module is 15 x 15. The discriminator and generator networks are updated alternately like in a standard GAN.”

### 4.2. Performance metrics

These three metrics Mean Squared Error (MSE), Precision, Recall, and Accuracy, along with Peak Signal-to-Noise Ratio (PSNR) and Structural Similarity (SSIM)—are used to quantitatively assess picture dehazing methods on synthetic images. Without ground-truth reference images, real-world images evaluate performance subjectively and visually. Traditional theory bases these measurements on the outcomes of binary classification, including true positive (TP), true negative (TN), false positive (FP), and false negative (FN). TP stands for successfully discovered, while TN represents correctly recognized elements. FP and FN indicate incorrectly recognized elements. These performance metrics are further described in the following paragraphs.

Precision: Precision is quantified as the percentage of accurately classified cases. To demonstrate this, utilize Equation (30).


$$\mathrm{Pr}ecision=\frac{TP}{TP+FP}$$ (30)

Recall: The term recall rate or recall may also refer to the genuine positive rate. It evaluates the frequency at which a classifier produces a good outcome for the correct category. Equations (31) are used to explain it.


Recall=TPTP+FN (31)

Accuracy: The total number of true positives and negatives is divided by all values to determine our forecasted value’s accuracy (32).


$$Accuracy=\frac{TP+TN}{TP+TN+FP+FN}$$ (32)

Mean square error (MSE): The mean square error is a commonly used metric in statistics and machine learning to determine the average squared difference between expected and actual data. The MSE formula is as follows:


$$MSE=\frac{1}{n}{\sum_{i=1}^{n}\left(Y_{i}-{\hat{Y}}_{i}\right)}^{2}$$ (33)

The number of data points in the dataset is indicated by n.

The actual (observed) value for the ith data point is represented by $Y_{i}$.

The character ${\hat{Y}}_{i}$ indicates the expected value for the ith data point.

∑ Signifies the sum of all data points between i = 1 and n.

Peak-signal-to-noise ratio (PSNR): It is the peak error measured and calculated as


$$PSNR=20\;\log_{10}\left(\frac{MAX_{F}}{\sqrt{MSE}}\right)$$ (34)

Pi is the likelihood that a pixel in imagine F would experience intensity i.

Structure similarity index measure (SSIM): SSIM analyzes the brightness, contrast, and structure of the improved patches at x and y locations to the original picture patches to determine whether they are the same.


$$SSIM(F,E)=\frac{\left(2\mu_{x}\mu_{y}+C_{1}\right)\left(2\sigma_{xy}+C_{2}\right)}{\left(\mu_{x}^{2}\mu_{y}^{2}+C_{1}\right)\left(\sigma_{x}^{2}\sigma_{y}^{2}+C_{2}\right)}$$ (35)

Where the standard deviation values of the pixels in patches x and y are $\sigma_{x}$, $\sigma_{y}$, and the mean values are $\mu_{x}$, $\mu_{y}$, correspondingly. The covariance of patches x and y is called $\sigma_{xy}$, and small constants C1 =  (k1L) 2 and C2 =  (k2L) 2 prevent instability when the denominator is near zero. L is the dynamic range of the pixel values, where k1 =  0.01 and k2 =  0.03. The greater the SSIM value, the less distortion there is, and the better the improvement.

### 4.3. Precision analysis

**Fig 9:**
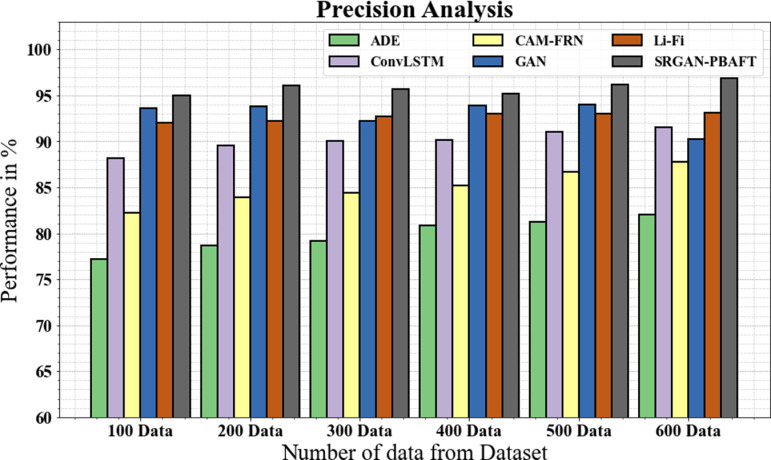
Precision analysis for SRGAN-PBFPT technique.

In Fig 9 and Table 1, the precision of the SRGAN-PBFPT methodology is contrasted with other commonly applied techniques. The improved precision performance of the deep learning method is illustrated in the graph where the SRGAN-PBFPT model’s precision for 100 data is 94.99%, while the ADE, ConvLSTM, CAM-FRN, GAN, and Li-Fi models have respective precisions of 77.19%, 88.19%, 82.19%, 93.55%, and 91.99%. Like this, the suggested SRGAN-PBAFT model has a precision of 96.88% under 600 data, compared to 81.99%, 91.52%, 87.77%, 95.19%, and 93.15% for the ADE, ConvLSTM, CAM-FRN, GAN, and Li-Fi models, correspondingly.

**Table 1:** Precision analysis for SRGAN-PBFPT technique.

No of data from Dataset	ADE	ConvLSTM	CAM-FRN	GAN	Li-Fi	SRGAN-PBAFT
100	77.19	88.19	82.19	93.55	91.99	94.99
200	78.67	89.56	83.88	93.78	92.19	96.11
300	79.13	89.99	84.44	92.19	92.67	96.66
400	80.88	90.18	85.17	93.87	92.98	95.99
500	81.25	90.99	86.66	93.99	92.99	96.14
600	81.99	91.52	87.77	90.19	93.15	96.88

### 4.4. Recall analysis

**Fig 10:**
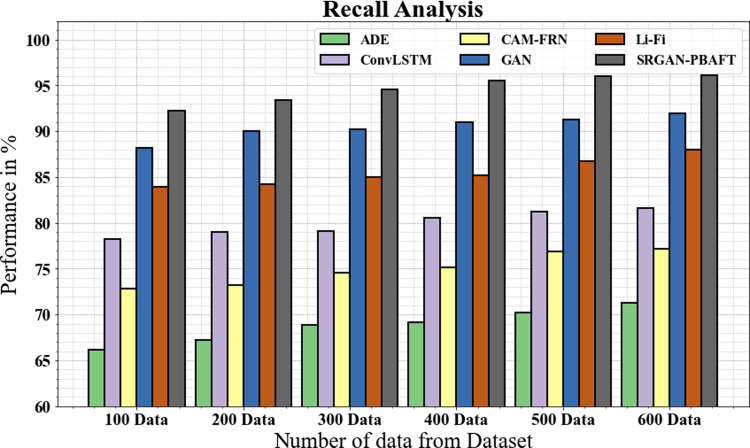
Recall analysis for SRGAN-PBAFT technique.

The recall of the SRGAN-PBAFT approach is compared to other frequently used techniques in Fig 10 and Table 2. The improved recall performance of the DL approach is depicted in the graph where the SRGAN-PBAFT model’s recall for 100 data is 92.19%, while the ADE, ConvLSTM, CAM-FRN, GAN, and Li-Fi models have recalls of 66.19%, 78.19%, 72.87%, 88.18%, and 83.98%, respectively. Like this, the suggested SRGAN-PBAFT model has a recall of 96.13% under 600 data, compared to 71.25%, 81.66%, 77.13%, 91.99%, and 87.99% for the ADE, ConvLSTM, CAM-FRN, GAN, and Li-Fi models, correspondingly.

**Table 2:** Recall analysis for SRGAN-PBAFT technique.

No of data from dataset	ADE	ConvLSTM	CAM-FRN	GAN	Li-Fi	SRGAN-PBAFT
100	66.19	78.19	72.87	88.18	83.98	92.19
200	67.19	78.99	73.19	89.99	84.18	93.44
300	68.88	79.13	74.55	90.17	84.99	94.55
400	69.19	80.56	75.14	90.99	85.19	95.55
500	70.17	81.23	76.88	91.23	86.77	95.99
600	71.25	81.66	77.13	91.99	87.99	96.13

### 4.5. Accuracy analysis

**Fig 11:**
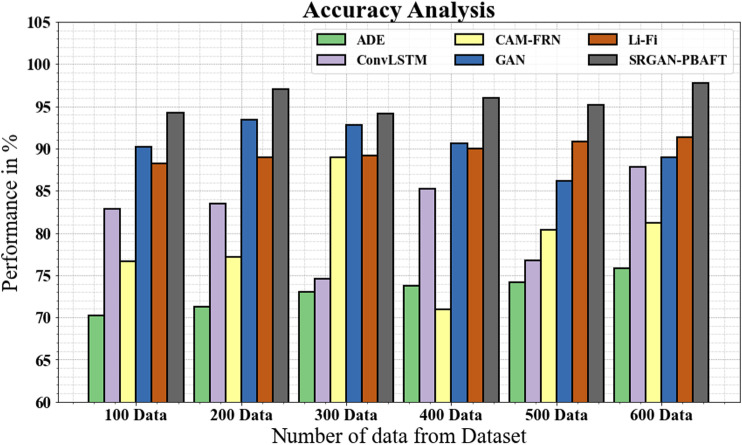
Accuracy analysis for SRGAN-PBAFT technique.

In Fig 11 and Table 3, the accuracy of the SRGAN-PBAFT strategy is compared with other commonly utilized techniques. The graph shows how the DL method has an enhanced accuracy performance. For instance, the accuracy with 100 data for the SRGAN-PBAFT model is 94.19%, compared to 70.19%, 82.88%, 76.66%, 92.19%, and 88.19% for the ADE, ConvLSTM, CAM-FRN, GAN, and Li-Fi models respectively. Similarly, the suggested SRGAN-PBAFT model achieves an accuracy of 97.99% with 600 data, compared to 75.78%, 87.77%, 81.23%, 96.99%, and 91.35% for the ADE, ConvLSTM, CAM-FRN, GAN, and Li-Fi models, respectively.

**Table 3:** Accuracy analysis for SRGAN-PBAFT technique.

No of data from dataset	ADE	ConvLSTM	CAM-FRN	GAN	Li-Fi	SRGAN-PBAFT
100	70.19	82.88	76.66	90.19	88.19	94.19
200	71.23	83.44	77.13	93.44	88.99	96.99
300	72.99	74.56	88.99	92.77	89.14	94.13
400	73.77	85.18	79.99	90.66	89.99	95.99
500	74.19	76.78	80.34	86.17	90.78	95.18
600	75.78	87.77	81.23	88.99	91.35	97.99

### 4.6. MSE analysis

**Fig 12:**
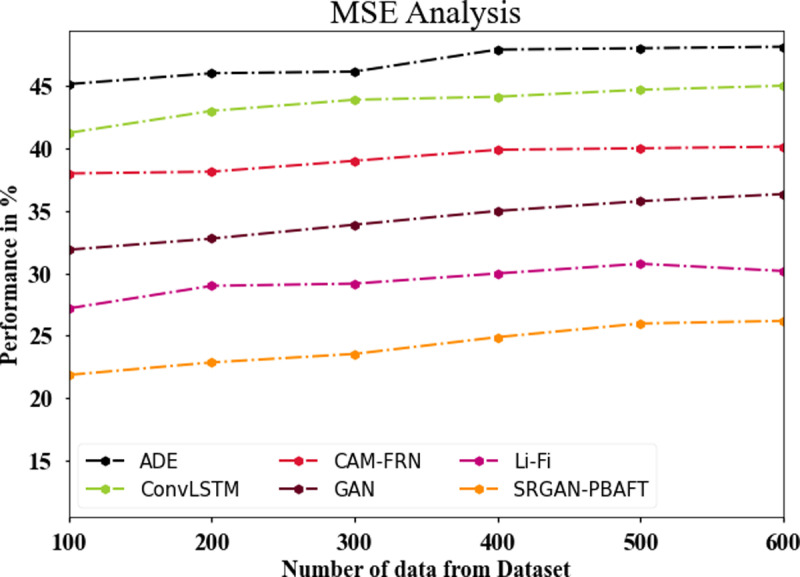
MSE analysis for SRGAN-PBAFT technique.

The MSE analysis of the SRGAN-PBAFT methodology with other techniques is presented in Fig 12 and Table 4. The graph illustrates how the DL method has an improved performance while reducing MSE. In contrast, the MSE values for the ADE, ConvLSTM, CAM-FRN, GAN, and Li-Fi models are 45.12%, 41.22%, 37.98%, 31.88%, and 27.19%, respectively, whereas the SRGAN-PBAFT model has an MSE of 21.87% with 100 data. The MSE value for the SRGAN-PBAFT model is 26.19% with 600 data, compared to 48.11%, 44.99%, 40.12%, 36.33%, and 30.18% for the ADE, ConvLSTM, CAM-FRN, GAN, and Li-Fi models, respectively.

**Table 4:** MSE analysis for SRGAN-PBAFT technique.

No of data from dataset	ADE	ConvLSTM	CAM-FRN	GAN	Li-Fi	SRGAN-PBAPT
100	45.12	41.22	37.98	31.88	27.19	21.87
200	45.99	42.98	38.12	32.77	28.99	22.87
300	46.12	43.87	38.99	33.88	29.17	23.55
400	47.88	44.11	39.87	34.98	29.98	24.89
500	47.99	44.67	39.99	35.76	30.76	25.98
600	48.11	44.99	40.12	36.33	30.18	26.19

### 4.7. PSNR analysis

**Fig 13:**
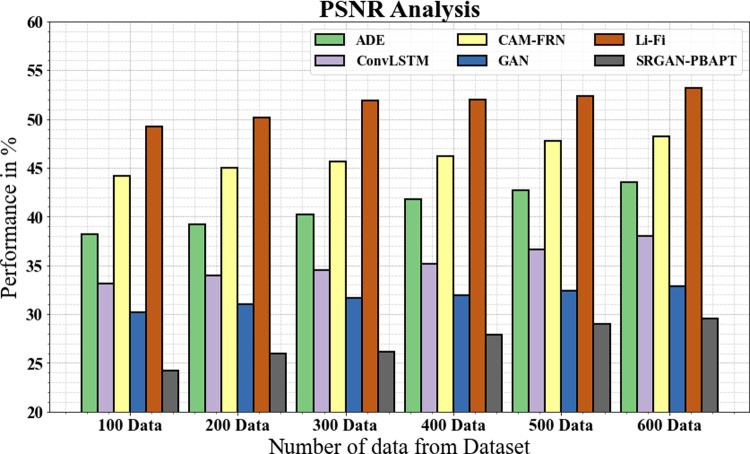
PSNR analysis for SRGAN-PBAFT technique.

The PSNR of the SRGAN-PBAFT approach is compared to other frequently used techniques in Fig 13 and Table 5. The graph shows how the DL method enhances PSNR performance. For instance, the PSNR for the SRGAN-PBAFT model is 24.19%, with 100 data compared to 38.19%, 33.18%, 44.16%, 30.18%, and 49.19% for the ADE, ConvLSTM, CAM-FRN, GAN, and Li-Fi models, respectively. Similarly, the suggested SRGAN-PBAFT model has a PSNR of 29.56% under 600 data, compared to 43.56%, 37.98%, 48.19%, 32.87%, and 53.19% for the ADE, ConvLSTM, CAM-FRN, GAN, and Li-Fi models, respectively.

**Table 5:** PSNR analysis for SRGAN-PBAFT technique.

No of data from dataset	ADE	ConvLSTM	CAM-FRN	GAN	Li-Fi	SRGAN-PBAFT
100	38.19	33.18	44.16	30.18	49.19	24.19
200	39.18	33.99	44.98	30.98	50.19	25.98
300	40.23	34.55	45.67	31.67	51.87	26.13
400	41.77	35.18	46.19	31.98	51.99	27.87
500	42.67	36.66	47.77	32.44	52.33	28.99
600	43.56	37.98	48.19	32.87	53.19	29.56

### 4.8. SSIM analysis

**Fig 14:**
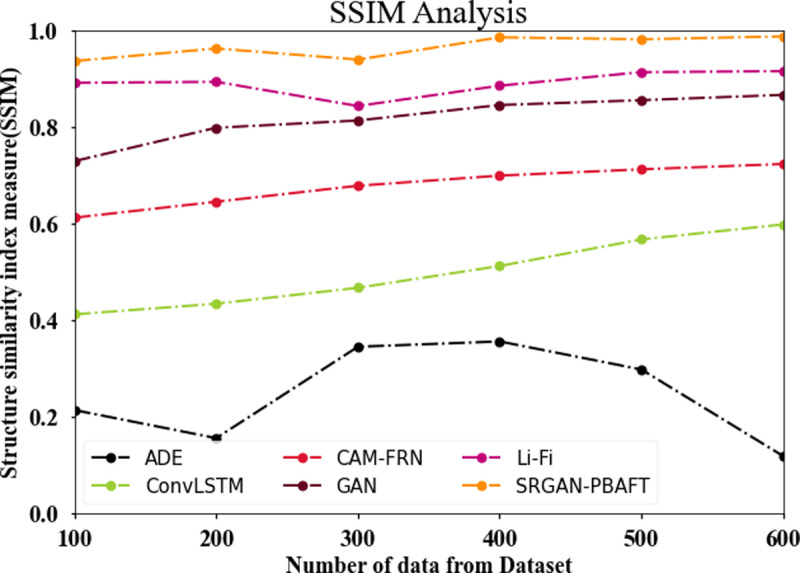
SSIM analysis for SRGAN-PBAFT technique.

In Fig 14 and Table 6, the SSIM of the SRGAN-PBAFT strategy is compared with other commonly used methods. The graph illustrates the improved SSIM performance of the DL approach. For instance, the SSIM with 100 data for the SRGAN-PBAFT model is 0.936%, compared to 0.214%, 0.412%, 0.612%, 0.729%, and 0.891% for the ADE, ConvLSTM, CAM-FRN, GAN, and Li-Fi models, respectively. Similarly, the suggested SRGAN-PBAFT model has an SSIM of 0.987% under 600 data, compared to 0.119%, 0.598%, 0.723%, 0.866%, and 0.915% for the ADE, ConvLSTM, CAM-FRN, GAN, and Li-Fi models, respectively.

**Table 6:** SSIM analysis for SRGAN-PBAFT technique.

No of data from dataset	ADE	ConvLSTM	CAM-FRN	GAN	Li-Fi	SRGAN-PBAFT
100	0.214	0.412	0.612	0.729	0.891	0.936
200	0.156	0.434	0.645	0.798	0.893	0.962
300	0.345	0.467	0.678	0.813	0.843	0.939
400	0.356	0.512	0.699	0.845	0.885	0.985
500	0.298	0.567	0.712	0.855	0.913	0.981
600	0.119	0.598	0.723	0.866	0.915	0.987

### 4.9. Ablation study

In the proposed model, every module is essential. In this section, we analyze the basis of the proposed SRGAN-PBAFT and current models such ADE, ConvLSTM, CAM-FRN, GAN, and Li-Fi by showing a series of ablation tests on the NYU2 Depth dataset, Middlebury stereo dataset, and SOTS dataset. Step-by-step addition of various features resulting by our proposed SRGAN-PBAFT model proves its reasons and helps study performance enhancement.

### 4.10. Influence of the PBAFT

**Fig 15:**
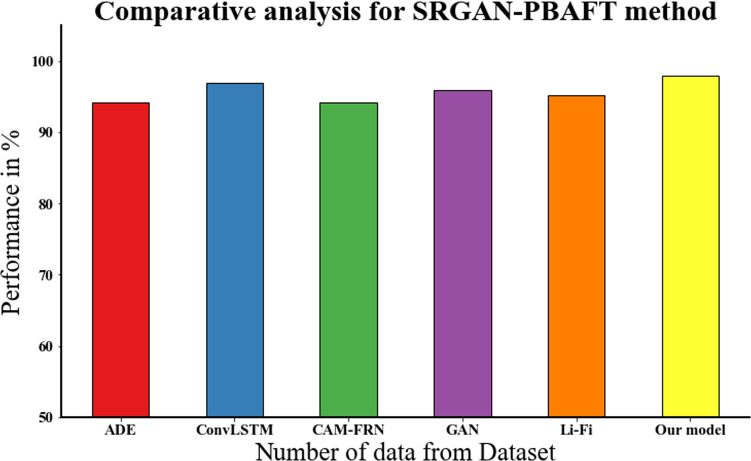
Comparative analysis for SRGAN-PBAFT method.

The accuracy analysis of the proposed SRGAN-PBAFT approach is compared to other existing methods shown in Fig 15 and Table 7. The PBAFT has a substantial impact in detecting and removing flickers from digital color images. By reasonably splitting images into smaller pieces, PBAFT professionally targets flickering spots, increasing its precision. It customizes its approach to each segment’s specific features using adaptive filtering techniques, ensuring excellent flicker removal while keeping visual details and color fidelity. PBAFT’s technique has low computing overhead, making it suitable for real-time applications. Finally, SRGAN combined with PBAFT model that achieved the superior performance of 97.99% for our input data. In assessment the existing MLP, CNN, SOM, DBN and GAN achieved accuracy performance of 94.19%, 96.99%, 94.13%, 95.99%, and 95.18% respectively.

**Table 7:** Comparative analysis for SRGAN-PBAFT method.

Methods	Accuracy
ADE	94.19
ConvLSTM	96.99
CAM-FRN	94.13
GAN	95.99
Li-Fi	95.18
Our model	97.99

### 4.11. Influence of the K-fold cross-validation

The 10-fold cross validation of the SRGAN-PBAFT approach is discussed in Table 8. By combining 10-fold cross-validation, the SRGAN-PBAFT-based flicker detection and removal method in digital color images is made much more dependable and robust. By splitting the dataset into 10 segments, or subsets, one is put sideways for validation and the other nine are used for training, a method of figures known as cross-validation is active. The suggested model SRGAN-PBAFT achieved a higher performance of 97.99% from our input data by using a 10-fold cross-validation procedure.

**Table 8:** SRGAN-PBAFT technique for 10-fold cross validation.

K-folds	SRGAN-PBAFT accuracy
1-Fold	0.93
2-Fold	0.94
3-Fold	0.96
4-Fold	0.95
Fold-5	0.96
Fold-6	0.94
Fold-7	0.97
Fold-8	0.98
Fold-9	0.98
Fold-10	0.97
10-Fold Mean	0.97

## 5. Conclusion

The combination of SRGANs-PBAFT method has produced a effective method for detecting and removing flickers in digital color images. SRGANs capably develop the awareness and simplicity of the images by addressing the problem of image resolution. The ability to achieve this outcome is enabled through significant enhancement in image quality for low-resolution inputs, resulting in the production of high-resolution outputs. Moreover, by training the generator network to produce representative textures and details, generative adversarial networks current a novel and reliable method to enhance the perceived quality of images. The study’s findings designate a respectable chance of improving the graphic quality of images with flickering artifacts. Our method offers a favorable solution to develop the excellence of digital color images and deliver users with a more immersive and aesthetically attractive experience as technology continues to advance. Future research could examine additional developments and additions to our suggested method, improving its performance and addressing more difficult problems in digital image improvement.
